# Purpose in Life and Loneliness Among African American Women: An Application of Latent Growth Mixture Modeling

**DOI:** 10.1093/geroni/igaf122.1764

**Published:** 2025-12-31

**Authors:** Eunbea Kim, Carolyn Cutrona, Daniel Russell

**Affiliations:** Auburn University, Auburn, Alabama, United States; Iowa State University, Ames, Iowa, United States; Iowa State University, Ames, Iowa, United States

## Abstract

Purpose in life has been widely proposed as a significant predictor of positive health outcomes. However, few studies have employed longitudinal methods to address the effects of purpose in life on loneliness. Furthermore, studies have rarely addressed the role of purpose in life in levels of loneliness among people of color. This study investigated the extent to which purpose in life predicted African American women’s loneliness over time. Using data from 661 African American women (Mage = 44.92, SD = 8.10) across four waves of the Family and Community Health Study (FACHS), latent growth mixture modeling was employed to explore the trajectories of loneliness across approximately 10 years and whether level of purpose in life was a significant predictor of the trajectories. This study also accounted for potent influential factors such as positive and negative social support, religiosity, racial discrimination, and financial strain as covariates. Findings revealed significant between-individual differences in loneliness trajectories, with individuals reporting a higher initial level of purpose in life tending to have lower levels of loneliness over time. Thus, greater purpose in life predicted lower loneliness among African American women, highlighting the importance of these factors in their psychosocial well-being.

